# MALDI-TOF MS: An effective tool for a global surveillance of dengue vector species

**DOI:** 10.1371/journal.pone.0276488

**Published:** 2022-10-20

**Authors:** Antsa Rakotonirina, Morgane Pol, Fara Nantenaina Raharimalala, Valentine Ballan, Malia Kainiu, Sébastien Boyer, Sosiasi Kilama, Sébastien Marcombe, Sylvie Russet, Emilie Barsac, Rama Vineshwaran, Malia Kaleméli Selemago, Vincent Jessop, Geneviève Robic, Romain Girod, Paul T. Brey, Julien Colot, Myrielle Dupont-Rouzeyrol, Vincent Richard, Nicolas Pocquet

**Affiliations:** 1 Institut Pasteur de Nouvelle-Calédonie, URE-Entomologie Médicale, Nouméa, New Caledonia; 2 Institut Pasteur de Madagascar, Unité d’entomologie Médicale, Antananarivo, Madagascar; 3 Institut Pasteur de Nouvelle-Calédonie, Groupe de Recherche en Bactériologie Expérimentale, Nouméa, New Caledonia; 4 Institut Pasteur du Cambodge, Medical and Veterinary Entomology Unit, Phnom Penh, Cambodia; 5 Department of Global Health, Institut Pasteur, Ecology & Emergence of Arthropod-borne Pathogens Unit, CNRS UMR2000, Paris, France; 6 Vector Borne Disease Laboratory, Institut Pasteur du Laos, Vientiane, Lao PDR; 7 Ministry of Health of Fiji, Suva, Fiji; 8 Agence De Santé Wallis et Futuna, Pole Prévention & Santé Publique, Uvea, Wallis & Futuna; 9 Institut Pasteur de Nouvelle-Calédonie, URE-Dengue et autres Arboviroses, Nouméa, New Caledonia; 10 Institut Pasteur, Direction Internationale, Paris, France; Fisheries and Oceans Canada, CANADA

## Abstract

Dengue, Zika and chikungunya viruses cause significant human public health burdens in the world. These arboviruses are transmitted by vector mosquito species notably *Aedes aegypti* and *Aedes albopictus*. In the Pacific region, more vector species of arboviruses belonging to the Scutellaris Group are present. Due to the expansion of human travel and international trade, the threat of their dispersal in other world regions is on the rise. Strengthening of entomological surveillance ensuring rapid detection of introduced vector species is therefore required in order to avoid their establishment and the risk of arbovirus outbreaks. This surveillance relies on accurate species identification. The aim of this study was to assess the use of the Matrix-Assisted Laser Desorption Ionization Time-Of-Flight Mass Spectrometry (MALDI-TOF MS) as a tool for an international identification and surveillance of these mosquito vectors of arboviruses. Field-mosquitoes belonging to 8 species (*Ae*. *aegypti*, *Ae*. *albopictus*, *Aedes polynesiensis*, *Aedes scutellaris*, *Aedes pseudoscutellaris*, *Aedes malayensis*, *Aedes futunae* and *Culex quinquefasciatus*) from 6 countries in the Pacific, Asian and Madagascar, were included in this study. Analysis provided evidence that a MALDI-TOF database created using mosquitoes from the Pacific region allowed suitable identification of mosquito species from the other regions. This technic was as efficient as the DNA sequencing method in identifying mosquito species. Indeed, with the exception of two *Ae*. *pseudoscutellaris*, an exact species identification was obtained for all individual mosquitoes. These findings highlight that the MALDI-TOF MS is a promising tool that could be used for a global comprehensive arbovirus vector surveillance.

## Introduction

The past 50 years have seen a dramatic emergence or re-emergence of arboviral diseases [1]. Their rapid worldwide spread has raised international concern. For example, chikungunya emerged from Africa in 2004, spread to the Indian Ocean islands and shortly after to Asia, Europe, Americas and the Pacific [2]. This is an acute febrile illness with sudden onset of fever and joint pains, particularly affecting the hands, wrists, ankles and feet [3]. Furthermore Zika fever emerged in the Pacific region and subsequently spread in several countries around the world leading to a massive outbreak in 2015–2016, especially in the Americas [4, 5]. This disease lead to dramatic consequences in pregnant women causing fetal brain damage and microcephaly [5]. In addition, dengue fever is endemoepidemic in most intertropical countries, and is responsible of 390 million infections and more than 9,000 deaths per year [6, 7].

These diseases are caused by arboviruses like chikungunya, Zika and dengue viruses (CHIKV, ZIKV and DENV) that are transmitted to humans through the bites of vector mosquitoes, mainly belonging to the genus *Aedes*, subgenus *Stegomyia*. The spread and the establishment of these vector mosquitoes are linked to the increase in urbanization, human travel, international trade and climate change [8–10]. The invasive mosquito *Ae*. *aegypti* represents one of the best examples of the global spread of vector species. This species is native to Africa and spread into the New World during the slave trade in the 15^th^ century [10]. This, subsequently spread into Asia in the 18^th^ century and into Europe and the Pacific region in the 20^th^ century [10, 11]. *Aedes albopictus*, native to Southeast Asia, is another widespread vector species. Its first expansion outside Asia was in the late of 1970s [10, 11] and was increased during the intercontinental trade in the 20^th^ century [12]. Despite its recent dispersal, this species is currently established in all continents, except Antarctica. These two species (*i*.*e*., *Ae*. *aegypti* and *Ae*. *albopictus*) are considered as the primary vectors of DENV, ZIKV, CHIKV [13–17] and could also transmit the Yellow fever virus (YFV) [18, 19].

The Pacific region encompasses a vast area containing more than 20,000 islands. To date, the main *Aedes* vectors of DENV, CHIKV and ZIKV of the Pacific region are *Ae*. *aegypti*, *Ae*. *albopictus* [13–17] and *Aedes polynesiensis* [20–22], these last two species belonging to the Scutellaris Group [23, 24]. In addition, at least nine other *Aedes* species of the Scutellaris Group present in the region are vectors, or highly suspected to be vectors of DENV such as *Ae*. *scutellaris* and *Ae*. *pseudoscutellaris* [23, 25]. Seven other species belonging to the Scutellaris Group are also documented in the Pacific region, but their potential implication in arboviruses transmission has not yet been established [24, 26]. Except for *Ae*. *albopictus* and *Ae*. *polynesiensis* which are widely distributed, some species of the Scutellaris Group are often highly localized [24]. For example, *Ae*. *futunae* is an endemic species of Futuna island [24]. However, globalization increases the risks of vectors dispersal and establishment in other Pacific islands, and in other world regions. This expansion risk highlights the need for improved entomological surveillance. This surveillance relies on accurate mosquito species identification, ensuring rapid detection of vector species introduced and prevention of their establishment through timely and adequate measures.

Morphological observations can be used to identify the *Aedes* species members of the Scutellaris Group [24] (https://www.wrbu.si.edu/vectorspecies/keys). However, the distinction of these similar species is very difficult. In addition, dichotomous keys currently used for their morphological identification need to be updated [24, 27, 28]. Their correct determination can also be compromised for specimens damaged during sampling.

DNA sequencing method is widely used to solve taxonomic and identification problems, but remains expensive and time-consuming. In addition, the pre-existing DNA sequence information is also often unavailable for some species of the Scutellaris Group, making comparison impossible.

Matrix-Assisted Laser Desorption Ionization Time-Of-Flight Mass Spectrometry (MALDI-TOF MS) has been more recently used for mosquito species identification [29–31]. This proteomic method generates protein spectra specific for each species [32]. The comparison of these spectra to a MALDI-TOF database composed of reference spectra allows to obtain a log score-value (LSV). This LSV is then compared to a threshold value to supports the accuracy of sample identification [29].

This method is faster than the DNA sequencing method. While the result of the identification of the mosquito species can be obtained in about a week for the DNA sequencing method, if sequencing is realized by outside supplier [29], the MALDI-TOF analysis can be performed in about 2.5 hours with experienced personnel [33]. In addition, despite the high initial cost of the MALDI-TOF machine, the costs of reagents and consumables required for MALDI-TOF analysis is lower than for DNA sequencing (1 Euro vs 20 Euro per sample, based on experience in New Caledonia) [29]. Moreover, this technique allowed reliable identification of mosquito species with distinction of specimens within a species complex (*i*.*e*. morphologically indistinguishable species) [30, 34] or from distant geographical areas. As an example, mosquito collected in Africa were species-identified by using a reference database created with mosquitoes collected from La Reunion Island [35]. Our recent work has underlined that the trapping duration, preservation condition and number of main spectrum pattern (MSPs) per species for the MALDI-TOF MS database creation must be optimized for mosquito species identification [29].

To our best knowledge, only one scientific article has investigated the use of MALDI-TOF to identify two of the major vectors of DENV in the Pacific region (*i*.*e*., *Ae*. *aegypti* and *Ae*. *polynesiensis*) using laboratory strain [36]. No other work was carried out regarding the use of this tool to distinguish the other *Aedes* species members of the Scutellaris Group from the field. In addition, the accuracy of a MALDI-TOF database created from specimens collected in the Pacific region to identify mosquitoes from other regions has never been evaluated. Therefore, taking into account the parameters previously described for the optimization of mosquito identification with MALDI-TOF MS, the aim of this study was to assess the use of the MALDI-TOF MS in surveillance of arboviruses vector species. For this purpose, our specific objectives were: (i) to assess whether the MALDI-TOF database, constituted of protein spectra obtained from legs of different mosquito species of the Pacific region, could be used to identify mosquitoes collected on a global scale and (ii) to evaluate the performance of this technique compared to the DNA sequencing method in identifying the *Aedes* species members of the Scutellaris Group.

## Methods

### Biological material

Adult and larvae mosquitoes used in this study were collected in six countries (Table 1). Three countries (Fiji, Wallis & Futuna, New Caledonia) are located in the Pacific region, two countries (Laos, Cambodia) in Southeast Asia and one country (Madagascar) in the African region. Six mosquito species included in this study are vectors of DENV: *Ae*. *aegypti*, *Ae*. *albopictus*, *Ae*. *malayensis*, *Ae*. *polynesiensis*, *Ae*. *pseudoscutellaris* and *Ae*. *scutellaris*. *Culex quinquefasciatus*, a cosmopolitan mosquito vector of other arboviruses was also analyzed. This species was not the focus of this study. It was included in order to test the MALDI-TOF effectiveness for international-scale identification. Field collections were undertaken from May 2018 to December 2020 except for *Ae*. *scutellaris*, collected on March 2016 and used in our previous work which has consisted of optimizing the use of the MALDI-TOF MS for mosquito species identification [29]. For adult collection, several sampling methods were used including the BG-sentinel® trap and BG-Gravid® *Aedes* trap (Biogents, Regensburg, Germany) and CDC light trap with CO_2_ (BioQuip, Compton, USA). As suggested by a previous study, traps were set in the field for a maximum of 24 h [29]. Larvae were collected in their breeding sites and reared to adults and then kept in -80C freezer for further analyses. Adults that emerged were harvested and killed. All mosquitoes were preserved on silica gel and cotton in the field and during the transport from the originating country to New Caledonia. When arrived in the laboratory, they were preserved at -80°C.

**Table 1:** Mosquitoes collected from each region.

Regions	Countries	Species	No. of mosquitoes used to create database	No. of mosquitoes used in blind testing
Pacific	Fiji	*Ae*. *aegypti*	5^#^	20
		*Ae*. albopictus*	6^#^	20
		*Ae*. pseudoscutellaris*	5^#^	20
		*Cx*. *quinquefasciatus*	5^#^	16
	Wallis	*Ae*. *aegypti*	-	4
		*Ae*. polynesiensis*	5^#^	20
		*Ae*. futunae*	5^#^	19
		*Cx*. *quinquefasciatus*	-	2
	New Caledonia [29]	*Ae*. scutellaris*	5^#^	9
		*Ae*. *aegypti*	10^#^	-
Asia	Cambodia	*Ae*. *aegypti*	5^+^	20
		*Ae*. albopictus*	3^+^	3
		*Cx*. *quinquefasciatus*	5^+^	19
	Laos	*Ae*. *aegypti*	5^+^	20
		*Ae*. albopictus*	5^+^	5
		*Ae*. malayensis*	2^+^	-
Africa	Madagascar	*Ae*. albopictus*	-	20
		*Cx*. *quinquefasciatus*	-	14
Total		-	71	231

* *Aedes* members of the Scutellaris Group.

^#^Specimens used in the Pacific database.

^+^ Specimens added to the Pacific database to generate the global database.

### Morphological and molecular identification of specimens

Before all experiments, mosquitoes were morphologically identified under stereo microscope using dichotomous identification keys [24, 27, 28]. Morphological identification was confirmed through double-blind evaluation by two experienced entomologists. All mosquitoes were subsequently dissected and for each specimen, legs were removed and transferred in 1.5 mL Eppendorf tube while the mosquito body was transferred in another tube.

Molecular analysis was realized for all the specimens used for the creation of the MALDI-TOF database. Since the species belonging to the Scutellaris Group are morphologically close, DNA sequencing was also assessed for those used in blind test analysis. For this, DNA was individually extracted from the body of each mosquito using a DNA Blood and Tissue Kit (Qiagen, Hilden, Germany) according to the manufacturer’s instruction. The extracted DNA was subsequently quantified using a NanoDrop 2000/2000c spectrophotometer (Thermo Scientific, Wilmington, USA) and amplified by polymerase chain reaction (PCR) using specific primers targeting the cytochrome *c* oxidase subunit 1 (*cox*1) [37]. Primers targeting the internal transcribed spacer 2 (ITS2) region of the ribosomal RNA gene cluster were also used for the molecular identification of the species members of the Scutellaris Group [38]. The PCR conditions were: 0.2 μM forward primer; 0.2 μM reverse primer; 1x master mix (Qiagen); distilled water; and template DNA (between 10 ng/μl and 100 ng/μl). The PCR was performed in a total volume of 25 μl under different thermal conditions according to the primers used. To amplify *cox*1, cycling involved an initial denaturation at 94°C for 3 min, 35 cycles of 94°C for 1 min, 50°C for 1 min and 72°C for 1 min, followed by a final extension step at 72°C for 10 min. To amplify ITS2, the thermal conditions were: 5 min at 95°C, 35 cycles of 1 min at 95°C, 1 min at 50°C and 90 s at 72°C, and a final extension step at 72°C for 10 min.

PCR Products were revealed on 1.5% agarose gel and those products showing unambiguous bands were processed for sequencing. The sequences obtained (Genoscreen, Lille, France) were assembled with PREGAP and GAP software (version 4.10.2, 2019; The GAP Group, GAP-Groups, Algorithms and Programming, Aachen, Germany) and compared with mosquito sequences available in the GenBank database using the BLAST platform (http://blast.ncbi.nlm.nih.gov/Blast.cgi).

### Phylogenetic analysis

Phylogenetic analysis was carried out for the *Aedes* species members of the Scutellaris Group. Mosquitoes belonging to the *Ae*. *aegypti* species were included in the analysis as outgroup taxa. During the study, a total of 49 *cox*1 nucleotide sequences were used in analysis, among which 40 *cox*1 sequences were generated in the current work and nine *cox*1 sequences were retrieved from the GenBank database. In addition, 49 ITS2 nucleotide sequences were also analyzed including 45 ITS2 sequences generated during this study and four ITS2 sequences from the GenBank database. Multiple sequence alignment of these sequences was achieved with Clustal W 1.7 integrated in Bioedit software (version 7.2.5). A maximum likelihood phylogenetic analysis was subsequently assessed using the MEGA software (version 10.1.7). The evolutionary distances were computed using the General Time Reversible methods, defined as the best evolutionary method with MEGA for the *cox*1 and the concatenated data sets. Regarding the ITS2 dataset, the Kimura 2-parameter model was used. For all analyses, support for internal nodes was estimated using the nonparametric bootstrap method with 1000 replications.

### Protein extraction for MALDI-TOF analysis

Mosquito proteins were extracted using legs, according to the protocol previously described [29]. To create the reference main spectrum pattern (MSP), eight spots of the same sample were deposited on the plate. Conversely, each sample intended to be queried against these reference spectra were deposited in duplicate, as suggested by previous work [29]. To control matrix quality (*i*.*e*. absence of MS peaks due to matrix buffer impurities), the matrix solution was loaded in duplicate onto each MALDI-TOF plate.

### MALDI-TOF MS parameters and analysis

The setting parameters of the MALDI-TOF were identical to those previously used [29]. Protein mass profiles were obtained using a Microflex MALDI-TOF mass spectrometer (Bruker, Wissembourg, France) within a mass range of 2,000–20,000 Daltons. The mass/charge ratio (m/z) of mosquito proteins is measured as it crosses an electromagnetic field after propulsion of the protein molecules by an ultraviolet laser. The measurement of this m/z ratio generated a specific protein mass spectrum for the body part of the considered species. Each spectrum corresponds to ions obtained from 240 laser shots performed in six regions of the same spot and automatically acquired using the Flex Control software, version 2.4 (Bruker Daltonics).

As previously stated, for each reference sample used to create MSP, the 8 replicates were individually measured 3 times in order to obtain 24 spectra [29]. Their MS spectra profiles were firstly visualized using MALDI flexAnalysis software, version 3.3 (Bruker Daltonics) to control MS spectra reproducibility. Then, a selection of 20–24 high quality spectra per sample were imported into the MALDI Biotyper compass software, version 3.1 (Bruker Daltonics) and used to create the MSP.

Conversely, one measurement per spot was realized for the sample used during the blind testing. The mass spectrum obtained was compared to the MSPs with the MALDI Biotyper compass software. This software calculates a log-score value (LSV) ranging from zero to three, reflecting the similarity between sample spectra and MSP. The Biotyper software showed the 10-best matching MSPs in an identification ranking list ordered according to the LSVs of each identification. For each sample analyzed, as previously suggested, the highest scoring spectrum was considered within the duplicate in order to improve the identification score [29]. In addition, during previous analysis, this is also the method classically used for mosquito species identification [39, 40].

According to the manual user of the MALDI Biotyper compass software, when two best matching MSPs belonged to two different species, the result was considered as doubtful.

### Creation and validation of Pacific and Asian MALDI-TOF MS database

Main spectrum pattern created during our previous work, consisting of 10 *Ae*. *aegypti*-MSPs and 5 *Ae*. *scutellaris* MSPs from New Caledonia were used [29]. Other MSPs were also created during this current study. These were created from 31 mosquitoes belonging to six species collected from other Pacific islands (Fiji and Wallis). All these MSPs formed the Pacific region database. In a second step, 25 other MSPs were created from Asian mosquitoes belonging to four species (Table 1). These formed the Asian region database. According to previous work, at least five MSPs per species were created [29]. Cluster analyses (MSP dendrogram) based on comparison of the MSPs were performed for the *Aedes* members of the Scutellaris Group.

Spectra of 222 mosquitoes from the Pacific, Asia and Africa regions created during this work were tested against the MALDI-TOF database. The spectra of nine *Ae*. *scutellaris*, a mosquito species recently introduced in New Caledonia were also included during the blind test analysis. These were created during our previous study [29].

### Revision of the threshold value for the Scutellaris Group

During this study, analyses were also carried out in order to determine the threshold value that allows correct differentiation between mosquito species belonging to the Scutellaris Group. For this purpose, field specimens including *Ae*. *albopictus*, *Ae*. *polynesiensis*, *Ae*. *scutellaris*, *Ae*. *pseudoscutellaris* and *Ae*. *futunae* were used. In order to test the MALDI-TOF accuracy when the MSPs of the species is not represented in the database, all mosquitoes belonging to these five species were first blinded queried against the MSPs for each species individually. These were also queried against MSP for a random selection of three species on the one hand, and of the other two species on the other hand. For these analyses, sensitivity (Se) and specificity (Sp) were computed as the rates of correctly classified spectra in the positive and negative labelled classes, respectively. These were calculated at various threshold values, ranging from zero to three. The positive predictive value (PPV), negative predictive value (NPV) and classification accuracy were also determined. The Receiver Operating Characteristics (ROC) curve was established. For this, Area Under Curve (AUC) values were evaluated to assess the discrimination of the MALDI-TOF MS analysis compared with the reference technique (*i*.*e*. DNA sequencing).

### Statistical analysis

Data were analyzed using R software (R Core Team (2017). R: A language and environment for statistical computing. R Foundation for Statistical Computing, Vienna, Austria). A non-parametric Wilcoxon test was carried out to compare LSV medians. Results with a two-sided p-value ≤ 0.05 were scored as being significant. The ROC analysis and graphs were also performed with R software.

## Results

### Morphological identification and molecular validation

The mosquitoes used in this study were classified morphologically into eight distinct species: *Ae*. *aegypti* (n = 79), *Ae*. *albopictus* (n = 62), *Ae*. *polynesiensis* (n = 25), *Ae*. *pseudoscutellaris* (n = 25), *Ae*. *malayensis* (n = 2), *Ae*. *scutellaris* (n = 14), *Ae*. *futunae* (n = 24) and *Cx*. *quinquefasciatus* (n = 61). Six of these species belonged to the Scutellaris Group: *Ae*. *albopictus*, *Ae*. *polynesiensis*, *Ae*. *pseudoscutellaris*, *Ae*. *malayensis*, *Ae*. *scutellaris* and *Ae*. *futunae*.

DNA sequencing was carried out in order to validate the morphological identification. A total of 266 sequences were generated (GenBank accession numbers: MW662168 to MW662252 and MW664678 to MW664858). The query of *cox*1 gene and ITS2 sequences against the GenBank database allowed to obtain reliable mosquito species identification for all the mosquito species of which reference sequences were available (Table 2). No *cox*1 sequences were available for *Ae*. *polynesiensis*, *Ae*. *pseudoscutellaris* and *Ae*. *futunae* in the GenBank database. No ITS2 sequences were also available for *Ae*. *scutellaris*, *Ae*. *malayensis* and *Ae*. *futunae*.

**Table 2:** Overview of mosquito identification by *cox*1 and ITS2 molecular typing.

Morphological identification	Species identified *via* GenBank database	Range of *cox*1 sequence similarity (%)	Range of ITS2 sequence similarity (%)
*Ae*. *aegypti*	*Ae*. *aegypti*	99–100	98–100
*Ae*. *albopictus*	*Ae*. *albopictus*	99–100	98–99
*Ae*. *scutellaris*	*Ae*. *scutellaris*	99	na
*Ae*. *malayensis*	*Ae*. *malayensis*	100	na
*Ae*. *polynesiensis*	*Ae*. *polynesiensis*	na	99
*Ae*. *pseudoscutellaris*	*Ae*. *pseudoscutellaris*	na	99–100
*Ae*. *futunae*	na	na	na
*Cx*. *quinquefasciatus*	*Cx*. *quinquefasciatus*	98–100	not sequenced

*Abbreviation*: na: not available; *cox*1: cytochrome c oxidase subunit 1; ITS2: internal transcribed spacer 2.

### Accuracy of the MALDI-TOF Pacific region database for mosquito species identification

**Fig 1:**
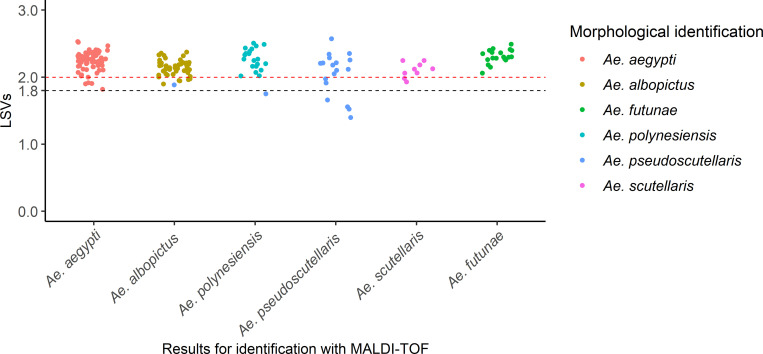
Comparison of the results obtained with morphological identification and MALDI‑TOF analysis for the *Aedes* species. X‑axis corresponds to the results obtained with MALDI‑TOF analysis. Y‑axis corresponds to Log-score values (LSVs). Colors illustrate morphological identification obtained through double‑blind evaluation and confirmed with DNA sequencing for the *Aedes* of the Scutellaris Group. Black dashed-line corresponds to the threshold value for mosquito species identification, stated during previous study [29]. Red dashed-line corresponds to the threshold value for the identification of mosquito species belonging to the Scutellaris Group.

Examples of spectrum of each mosquito species are provided in the supplementary file (S1 Fig). The spectra of 231 mosquitoes collected from the three regions were firstly queried against the Pacific region database created during this study. The raw results are shown in the supplementary file (S1 Table). The result showed reliable identification for 96% (95% CI: 87–99%) of *Cx*. *quinquefasciatus* mosquitoes, with LSVs greater than or equal to the threshold of 1.8 retained for mosquito species identification [29, 30]. In addition, 95% (95% CI: 90–98%) of *Aedes* mosquitoes were also correctly identified with LSVs greater than or equal to the 1.8 (Fig 1).

Among the 182 *Aedes* mosquitoes tested, two *Ae*. *pseudoscutellaris* were not correctly identified. These were identified as *Ae*. *polynesiensis* (LSV = 1.75) and *Ae*. *albopictus* (LSV = 1.88), respectively (Fig 1). However, their top two matching MSPs belonged to two different mosquito species. In addition, four *Aedes* of the Scutellaris Group (*i*.*e*. three *Ae*. *pseudoscutellaris* and one *Ae*. *polynesiensis*) also showed a discordance between the species of their top two matching MSPs.

**Fig 2:**
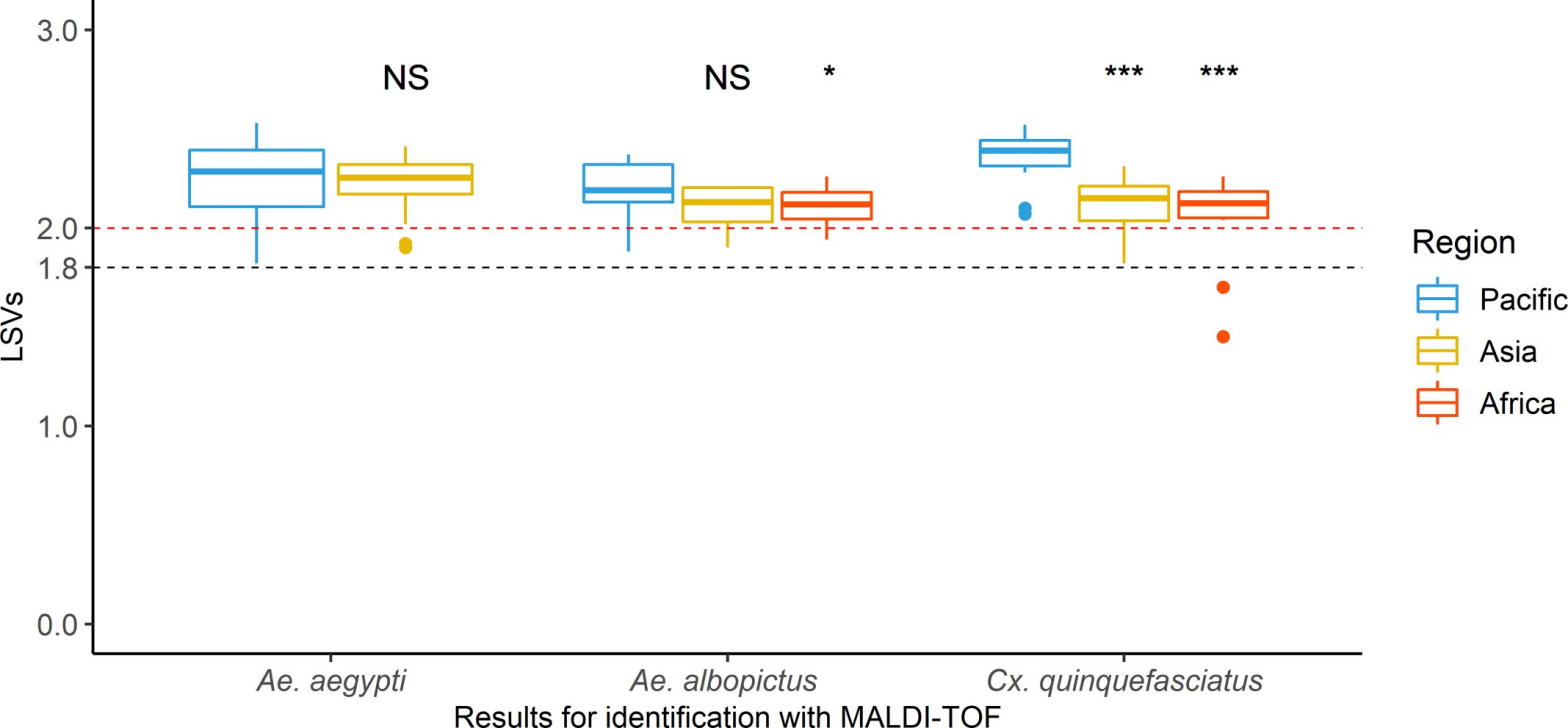
Identification scores of mosquitoes collected from the three regions when using the Pacific region MALDI-TOF database. Three mosquito species were analyzed: *Aedes aegypti*, *Aedes albopictus* and *Culex quinquefasciatus*. Colors illustrate the region of collection. Each boxplot consists of nine to 40 LSVs per species and per region. Black dashed-line corresponds to the threshold value for mosquito species identification, stated during previous study [29]. Red dashed-line corresponds to the threshold value for the identification of mosquito species belonging to the Scutellaris Group. Wilcoxon test, *p-value < 0.05, ***p-value < 0.001. Abbreviation: NS, not significant.

A second analysis was performed in order to compare LSVs obtained for the Pacific mosquitoes to those collected from the Asian and African regions when using Pacific region database only. Three cosmopolitan species *Ae*. *aegypti*, *Ae*. *albopictus* and *Cx*. *quinquefasciatus* were used (Fig 2). The result showed high LSVs for all the mosquitoes analyzed. Although the significant difference between LSVs median of *Ae*. *albopictus* from the Pacific islands and those from Madagascar (2.13 and 2.12, respectively; Wilcoxon test, p-value = 0.01), high LSV median were obtained. A significant difference was also observed between *Cx*. *quinquefasciatus* from the Pacific and Asian regions (2.39 and 2.15, respectively; Wilcoxon test, p-value = 6.3 x 10^−6^) and between those from the Pacific and African regions (2.39 and 2.12, respectively; Wilcoxon test, p-value = 4.4 x 10^−5^). However, LSVs median remained above 2 (Fig 2).

The results only showed a slight increase of LSVs medians for *Ae*. *aegypti* (from 2.26 to 2.3, Wilcoxon test, p-value = 0.009) and *Cx*. *quinquefasciatus* (from 2.2 to 2.24, Wilcoxon test, p-value = 0.04) when they were compared with the global database including Pacific and Asian MSPs (S2 Fig). Conversely, no significant difference was observed between the LSVs median of *Ae*. *albopictus* regardless of the MSPs included in analysis (Wilcoxon test, p-value = 0.63).

### Threshold value for the mosquito species belonging to the Scutellaris Group

Other analyses were performed on species belonging to the Scutellaris Group, in order to test the accuracy of the threshold value of 1.8, set during previous studies. The optimal threshold values which maximized the MALDI-TOF MS sensitivity and specificity ranged from 1.87 to 2.01 when only one species was included in the MALDI-TOF database (Table 3). High PPV and NPV at these thresholds was obtained, except when only *Ae*. *pseudoscutellaris* MPSs were used. Indeed, for this species, at a threshold-value of 1.87, PPV of 0.38 was obtained, with 24 false-positive specimens (Table 3). Among these false-positives, 15 were *Ae*. *polynesiensis* mosquitoes and nine were *Ae*. *albopictus* mosquitoes.

**Table 3:** Overview of results obtained during the determination of the optimal threshold for *Aedes* of the Scutellaris Group identification with MALDI‑TOF MS.

Species in the database	Optimal threshold	TP	FN	TN	FP	Sensitivity	Specificity	PPV	NPV	AUC	CI_95_ (AUC)
*Ae*. *scutellaris*	1.92	9	0	107	0	1	1	1	1	1	[1–1]
*Ae*. *albopictus*	1.94	46	2	62	6	0.94	0.94	0.88	0.97	0.99	[0.97–1]
*Ae*. *polynesiensis*	2.01	20	0	94	2	1	0.98	0.91	1	0.99	[0.97–1]
*Ae*. *pseudoscutellaris*	1.87	15	5	72	24	0.7	0.75	0.38	0.93	0.77	[0.65–0.90]
*Ae*. *futunae*	1.93	19	0	97	0	1	1	1	1	1	[1–1]
3 species^(1)^	1.99	39	9	56	12	0.82	0.82	0.76	0.68	0.88	[0.81–0.96]
2 species^(2)^	1.99	63	5	46	2	0.93	0.96	0.97	0.90	0.98	[0.95–1]

^(1)^ *Ae*. *scutellaris*, *Ae*. *pseudoscutellaris* and *Ae*. *futunae*.

^(2)^ *Ae*. *polynesiensis* and *Ae*. *albopictus*.

Abbreviations: AUC, area under the curve; TP, true positives; FN, false negatives; TN, true negatives; FP, false positives; PPV, positive predictive values; NPV, negative predictive values. CI_95_, Confidence Intervals at 95%.

When approximately half the species of the Scutellaris Group were used in the database, the optimal threshold value was of 1.99 (*i*.*e*. approximately 2). PPV ranging from 0.76 to 0.97, NPV ranging from 0.68 to 0.9 and AUC values ranging from 0.88 to 0.98 were also observed (Table 3; S3 and S4 Figs).

### Phylogenetic analysis of *Aedes* members of the Scutellaris Group

**Fig 3:**
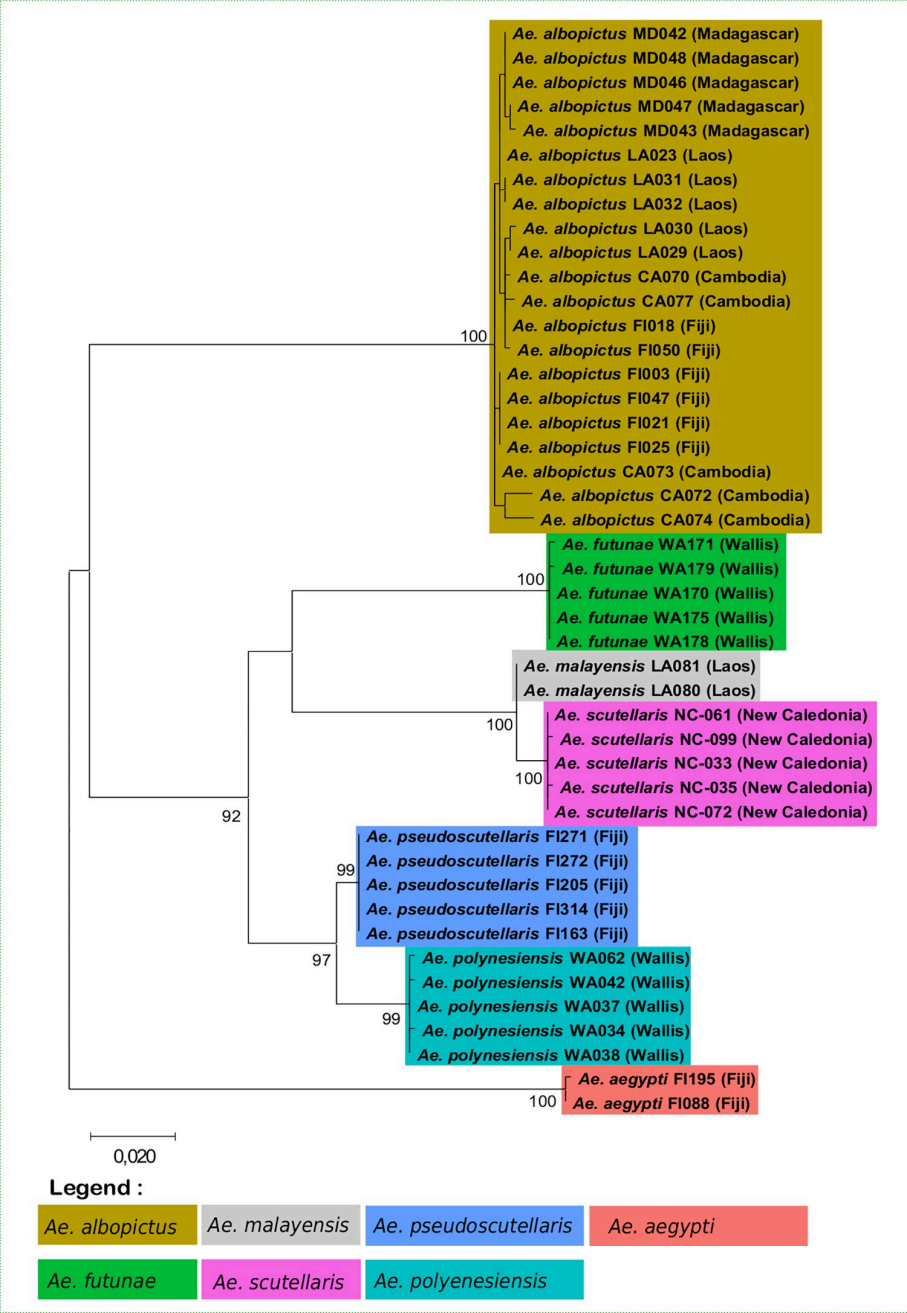
Maximum likelihood evolutionary (MLE) relationships of mosquito species of the Scutellaris Group. Tree derived from 45 concatenated sequences. For analysis, the percentage of replicate trees in which the associated taxa clustered together in the bootstrap test (1000 replicates) is shown for values over 80. The country of collection is indicated along with the specimen number. Colors indicate mosquito species.

The *cox*1 and ITS2 sequences of 616 and 234 nucleotides respectively obtained from the *Aedes* of the Scutellaris Group was aligned. The aligned and concatenated sequences from *cox*1 and ITS2 resulted in a block of 850 nucleotides. The result of phylogenetic analysis on these concatenated sequences showed that *Ae*. *albopictus* formed a well-supported monophyletic group, with 100% of bootstrap value (Fig 3), but without evidence for a distinction between geographic origin. The other species of the Scutellaris Subgroup were well separated when using the concatenated sequences.

**Fig 4:**
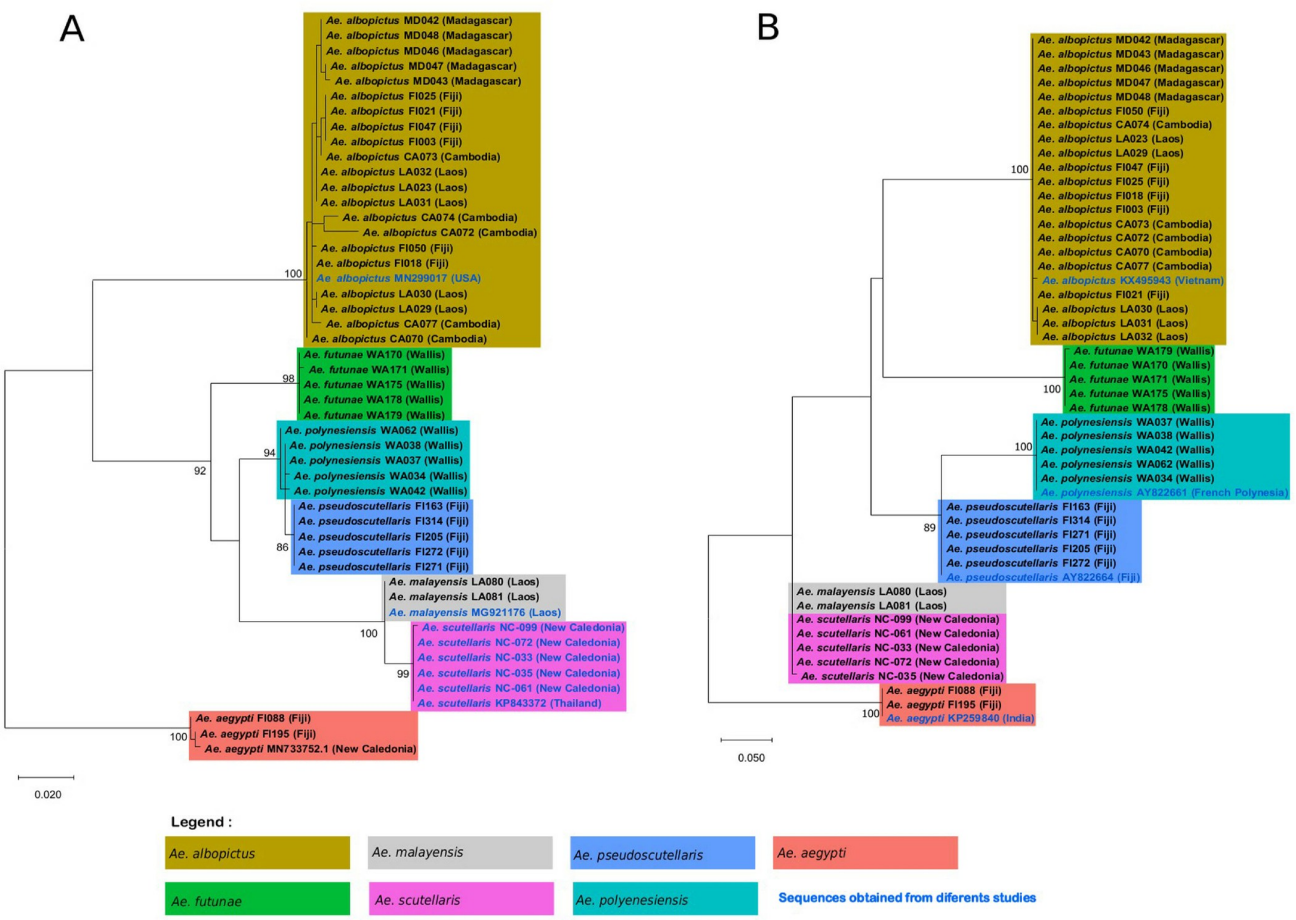
Maximum likelihood evolutionary (MLE) relationships of mosquito species of the Scutellaris Group. A—Maximum likelihood tree derived from 49 *cox*1 nucleotide sequences. Forty sequences generated during the current study (in black), six sequences generated during our previous study and deposited in the GenBank database (in black) [29] and three sequences retrieved from the GenBank database (in blue). B—Maximum likelihood tree derived from 49 ITS2 nucleotide sequences. Forty-five sequences were generated during this study (in black) and four sequences were retrieved from the GenBank database (in blue). For all analyses, the percentage of replicate trees in which the associated taxa clustered together in the bootstrap test (1000 replicates) is shown for values over 80. The country of collection is indicated along with the specimen number. Colors indicate mosquito species.

Interestingly, analysis based on the *cox*1 gene alone indicated few difference (*i*.*e*., three substitutions) between sequences of *Ae*. *polynesiensis* collected in Wallis and *Ae*. *pseudoscutellaris* collected in Fiji, resulting in a short evolutionary distance (Fig 4A). The evolutionary distance between these two species were, however, much greater when using the ITS2 sequences alone (Fig 4B). Conversely, no evolutionary distance was observed between *Ae*. *malayensis* and *Ae*. *scutellaris* when analyzing the ITS2 sequence (Fig 4B). Finally, the sister clade of *Ae*. *futunae* changed according to nucleotide sequences analyzed (i.e., *cox*1 or ITS2; Fig 4), but this species formed a single well-supported monophyletic group in both cases (*i*.*e*., bootstrap value ≥ 98).

**Fig 5:**
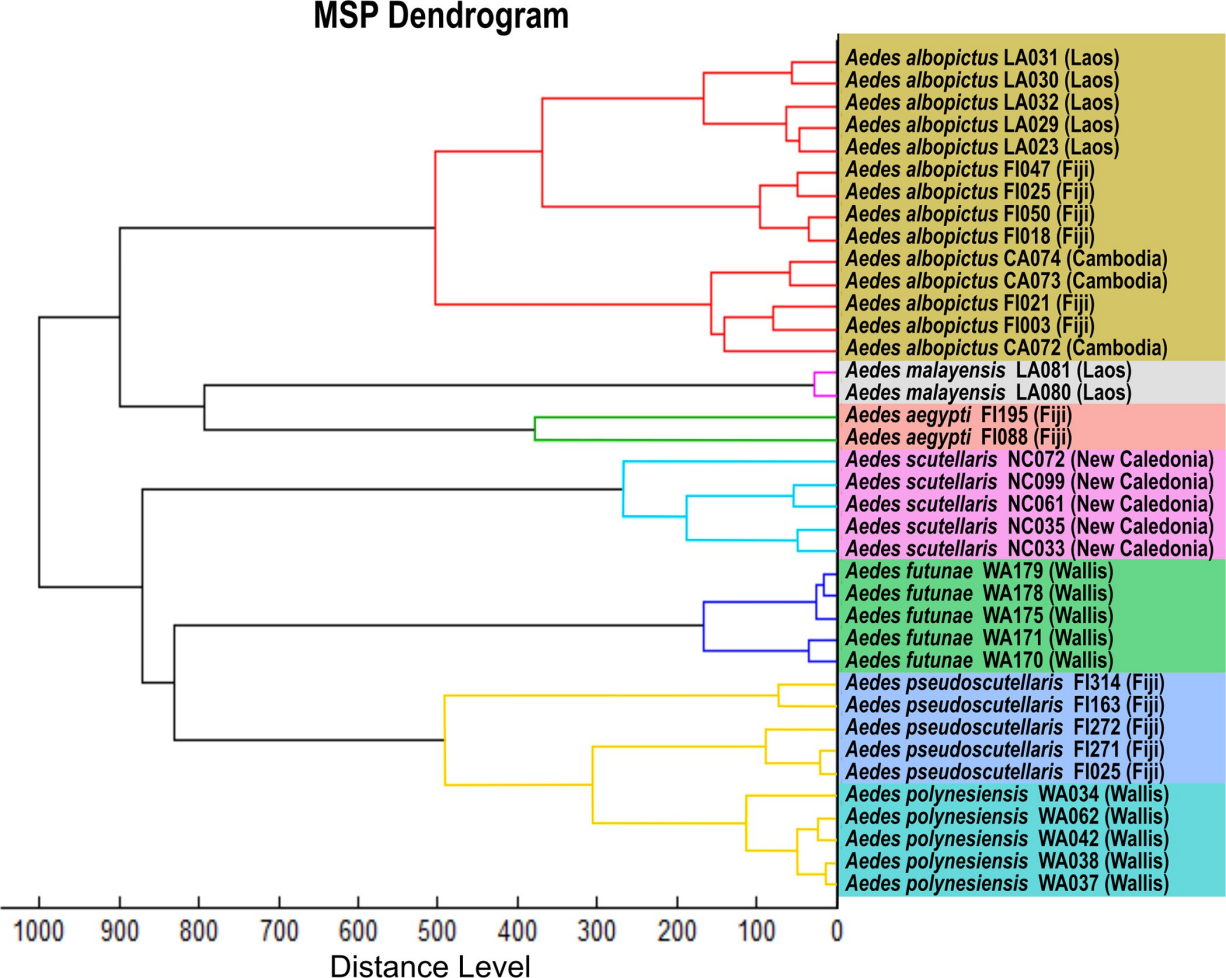
MSP dendrogram of all the mosquitoes belonging to the Scutellaris Group in the MALDI‑TOF database. The two *Aedes aegypti* was used as outgroup taxa. Colors of the branches indicate mosquito species according to the cut-off of 500 (arbitrary units) previously described [41].

A dendrogram was also created according to the protein mass profiles of the *Aedes* members of the Scutellaris Group. Specimens from the same species were clustered on the same branch and no species were intertwining with the exception of *Ae*. *polynesiensis* and *Ae*. *pseudoscutellaris* (Fig 5). Unlike for phylogenetic tree, much greater distances were observed between *Ae*. *malayensis* and *Ae*. *scutellaris*.

## Discussion

In this work, seven mosquito species from three islands in the Pacific region, including three important DENV vector species were initially used to create the MALDI-TOF database. The collected mosquitoes from the three regions showed that the Pacific region database allowed the species identification of all the specimens analyzed from Fiji, Wallis & Futuna (Pacific region), Cambodia, Laos (Asian region) and Madagascar (African region). Likewise, the difference of sampling sites between the mosquitoes used to create the database and those to be identified did not alter the accuracy of the MALDI-TOF for species identification [35]. In contrast in another study, leg spectra heterogeneity for *Anopheles gambiae* according to their geographical origins lead to high proportions of incorrect identifications when using a database without specimens from the same field collection [42]. Their results might have been impacted by the preservation method and duration of some specimens [42]. Indeed, mosquito preservation is one of the important parameters needed to be considered for a successful species identification with MALDI-TOF MS [29]. However, while considering optimized recommendations (*i*.*e*., the trapping duration, the preservation method and the number of MSPs per species in the database), MALDI-TOF MS is an efficient technique for the identification of mosquitoes collected from distant geographical regions.

In a second step, other MSPs were created using mosquito species from Asia including *Ae*. *aegypti*, *Ae*. *albopictus* and *Cx*. *quinquefasciatus*. During this analysis, a slight improvement of LSVs was found for *Ae*. *aegypti* and *Cx*. *quinquefasciatus* collected from Asia. This finding suggests that the addition in the database of specimens of the same origin may improve the identification result. A significant increase of LSVs was also found during a previous studies, when the database was upgraded with MSPs created from specimens of the same origin as the specimens to be tested [42]. In our case, however, high LSV medians were observed even when only MSPs from the mosquitoes collected in the Pacific region were used. Therefore, the MALDI-TOF database of the Pacific region could be sufficient to identify mosquito species from other regions.

During the analysis, *Ae*. *futunae* has been successfully distinguished from other species of group which are vectors of arboviruses. In addition, no mismatch was observed for the majority of the *Aedes* species of the Scutellaris Group, except for two *Ae*. *pseudoscutellaris*. These were misidentified as *Ae*. *polynesiensis* and *Ae*. *albopictus*, respectively. Some mismatches were also found for mosquitoes within a species complex during previous studies [34, 42]. In our case, for these two misidentified mosquitoes, the top two matching MSPs belonged to two different species of the Scutellaris Group. Likewise, four other *Aedes* of the Scutellaris Group (*i*.*e*., three *Ae*. *pseudoscutellaris* and one *Ae*. *polynesiensis*) showed mismatches between their top two corresponding MSPs species. These findings highlighted a possible proximity of protein profiling for the *Aedes* of the Scutellaris Group. The concordance between species of the top two matching MSPs could be a parameter to be check for precise species identification, as it is the case for identification of bacterial species in medical diagnosis. Thus, these mosquitoes could be considered as not identified by the MALDI-TOF MS.

These observations prompted us to revise the threshold value for the accuracy of the Scutellaris Group identification. The result showed an optimal threshold value greater than that determined during previous studies [29, 30] regardless of MSPs used during analysis. As previously supposed, the threshold value of 1.8 could depend on the richness of mosquito species diversity in the MALDI-TOF database [29]. Moreover, this threshold value could be insufficient to correctly distinguish mosquito species that are phylogenetically related. The result of this current study suggests that, for a secure identification score, a threshold value of 2 should be retained for the *Aedes* of the Scutellaris Group. During this revision of the threshold value, high PPV and NPV were obtained regardless the analysis, except when only *Ae*. *pseudoscutellaris* MSPs were present in the database. This low PPV obtained was due to the high number of false-positive specimens; the majority of which were *Ae*. *polynesiensis* mosquitoes. This finding also demonstrates that the spectra of the *Aedes* of the Scutellaris Group could have similarities, especially those of *Ae*. *pseudoscutellaris* and *Ae*. *polynesiensis*.

All the *Aedes* of the Scutellaris Group included in this study were sequenced using *cox*1 and ITS2 primers. The first *cox*1 sequences of *Ae*. *polynesiensis*, *Ae*. *pseudoscutellaris* and *Ae*. *futunae* and the first ITS2 sequences of *Ae*. *scutellaris*, *Ae*. *malayensis* and *Ae*. *futunae* were presented in this work. Phylogenetic analysis was subsequently assessed for the specimens used to create MSPs. Few sequence differences were observed between *Ae*. *polynesiensis* and *Ae*. *pseudoscutellaris* when using the *cox*1 gene. Controversial hypotheses are known concerning the taxonomic status of these two species. Due to the absence of reproductive barrier under experimental conditions, some authors have suggested that *Ae*. *polynesiensis* and *Ae*. *pseudoscutellaris* formed an unique species [43]. Other author has suggested that these are two distinct species recently separated [24]. In any case, our result showed that the *cox*1-based DNA barcoding, widely used for mosquito species identification, is not sufficient to distinguish *Ae*. *polynesiensis* to *Ae*. *pseudoscutellaris*. In contrast, the analysis of the ITS2 sequence allowed a distinction between these two species. Likewise, a previous study showed an important difference between these two species when using the ITS2 sequence [38].

During analysis, the results also indicated that *Ae*. *scutellaris* was phylogenetically closely related to *Ae*. *malayensis* with no phylogenetic distance when analyzing the ITS2 sequence. No phylogenetic distance was found between *Ae*. *scutellaris* and some species of the Scutellaris Group when using 16S ribosomal RNA and 28S ribosomal RNA genes during previous work [44]. These findings demonstrated that despite the increasing of the use of DNA sequencing in mosquito surveillance programs, this technique shows some limitations regarding the distinction between species of the Scutellaris Group.

In the dendrogram obtained with MALDI-TOF MS, each species of the Scutellaris Group clustered together, except for *Ae*. *polynesiensis* and *Ae*. *pseudoscutellaris*. Indeed, the separation was not clear for these two species in the MSP dendrogram. Nevertheless, all mosquitoes belonging to these two species were correctly distinguished during the blind test analysis, except for two *Ae*. *pseudoscutellaris*. Moreover, unlike for the phylogenetic trees, high distance level was observed between *Ae*. *malayensis* and *Ae*. *scutellaris*. Some authors have also found this discordance between results obtained with MSP dendrograms and phylogenetic trees for *Anopheles* species from French Guiana and Australian mosquito species [39, 40]. These observations confirmed other authors’ finding that MALDI-TOF MS cannot yet be used for phylogenetic analyses [39].

Overall, this work provides a methodology of great significance for the identification of mosquito vectors of arboviruses. The validation of our MALDI-TOF database at an international scale is one of the main strength of this study, besides the demonstration of its ability to accurately distinguish mosquito species which are morphologically close and barely separable using *Cox*1 gene.

This study may however suffer from biases and limitations. First, only six *Aedes* species of the Scutellari*s* Group were included in this database. About 40 species is currently classified in this group with at least 12 species vectors of DENV [23, 24, 26, 45]. However, the results obtained are very encouraging and constitute a proof of concept for using this technique to identify the other species of the Scutellaris Group. This work has also allowed to initiate a MALDI-TOF database containing mosquito species of public health importance in the Asia-Pacific region.

Secondly, only two MSPs were created for *Ae*. *malayensis*, a species vector of DENV and CHIKV [45], due to the insufficient number of specimen available. In addition, no mosquito belonging to this species was used for blind testing. As observed during previous study, at least five MSPs per species is required to optimize a MALDI-TOF database. Other *Ae*. *malayensis* should be added to the database and queried against MSPs.

## Conclusion

The MALDI-TOF MS provides an accurate and international-scale identification of mosquitoes even for morphologically and phylogenetically closely related species. Therefore, it can be used to reinforce the sequencing method for more rapid identification of introduced vector species, allowing rapid intervention of public health authorities. The inclusion of other vector mosquito species in this database is needed to use the MALDI-TOF MS as an effective method for global surveillance. In addition, the creation of a MALDI-TOF database platform is required for a collection and sharing of mosquito species spectra, especially for the neglected potentially invasive species like some *Aedes* of the Scutellaris Group. Finally, the assessment of the taxonomic status of these *Aedes* belonging to the Scutellaris Group, using a phylogenetic approach based on multiple genes is also necessary.

## Supporting information

S1 Fig Examples of spectrum of each mosquito species. (TIFF)

S2 Fig Comparison of Log-score values (LSVs) of Asian mosquitoes when using the Pacific MALDI-TOF database on the one hand the Asian-Pacific database on the other hand. Three mosquito species were analyzed: *Aedes aegypti* (n = 40), *Aedes albopictus* (n = 8) and *Culex quinquefasciatus* (n = 19). Colors illustrate the database used in analysis. Black dashed-line corresponds to the threshold value for mosquito species identification, stated during previous study [29]. Red dashed-line corresponds to the threshold value for the identification of mosquito species belonging to the Scutellaris Group. Wilcoxon test, *p-value < 0.05, **p-value < 0.01. Abbreviation: NS, not significant.
(PDF)

S3 Fig Determination of the threshold value of MALDI‑TOF MS for *Aedes* of Scutellaris Group identification when using *Aedes scutellaris*, *Aedes pseudoscutellaris* and *Aedes futunae* reference spectra in the MALDI-TOF database. A- Representation of the AUC (Area under curve). B–Representation of the threshold value which maximizes the sensitivity and specificity of the technique.
(PDF)

S4 Fig Determination of the threshold value of MALDI‑TOF MS for *Aedes* of Scutellaris Group identification when using *Aedes polynesiensis*, *Aedes albopictus* reference spectra in the MALDI-TOF database. A- Representation of the AUC (Area under curve). B–Representation of the threshold value which maximizes the sensitivity and specificity of the technique.
(PDF)

S1 Table Raw data showing the identification scores obtained for the duplicate of each mosquito. (XLSX)
