# Supplementary figures and images for: MALDI-TOF MS: An effective tool for a global surveillance of dengue vector species
Source: PLoS One. 2022 Oct 20;17(10):e0276488. doi: 10.1371/journal.pone.0276488 (PMC9584457; doi:10.1371/journal.pone.0276488)

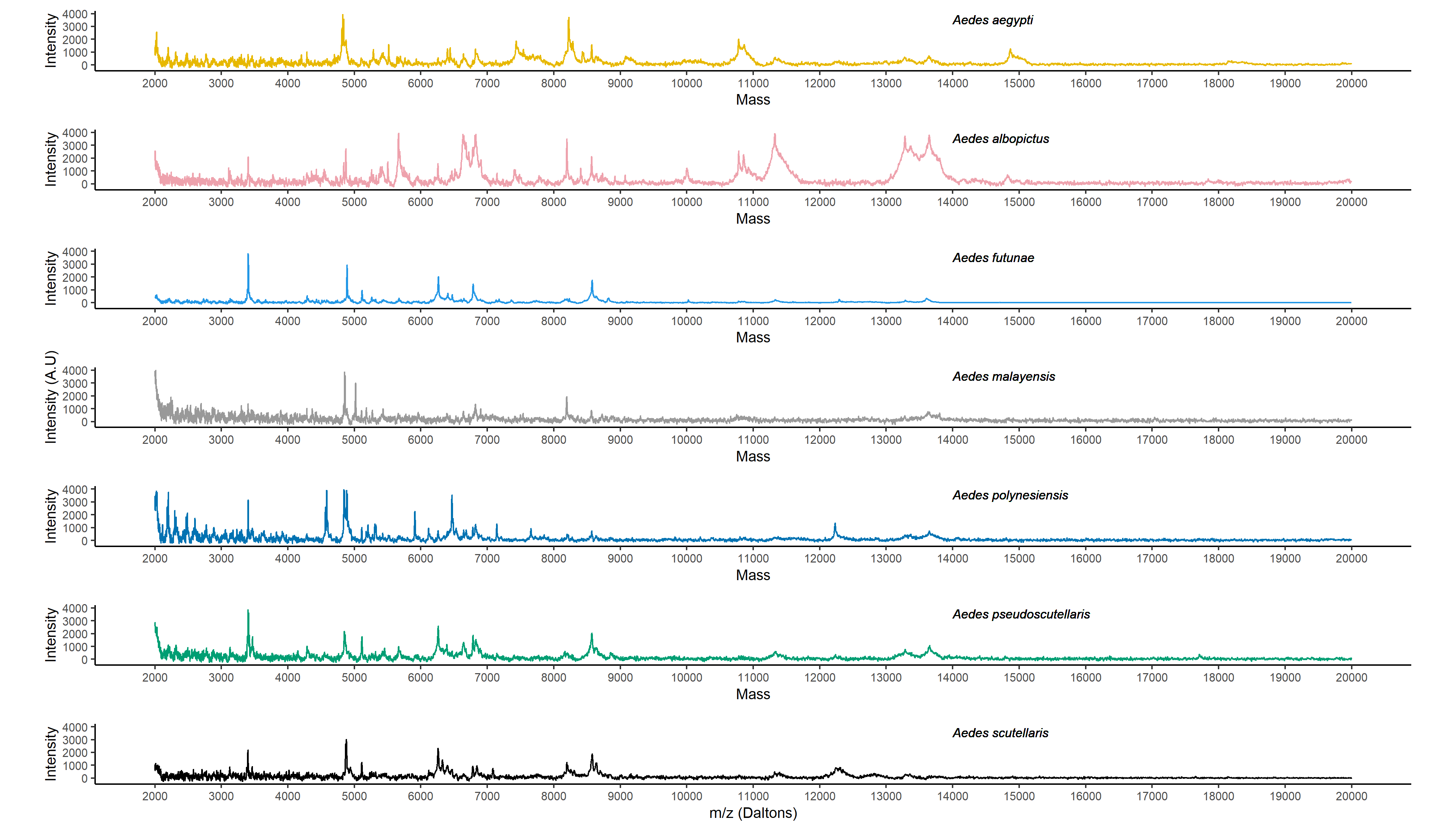

Supplement: S1 Fig — (TIFF) [file pone.0276488.s001.tiff]

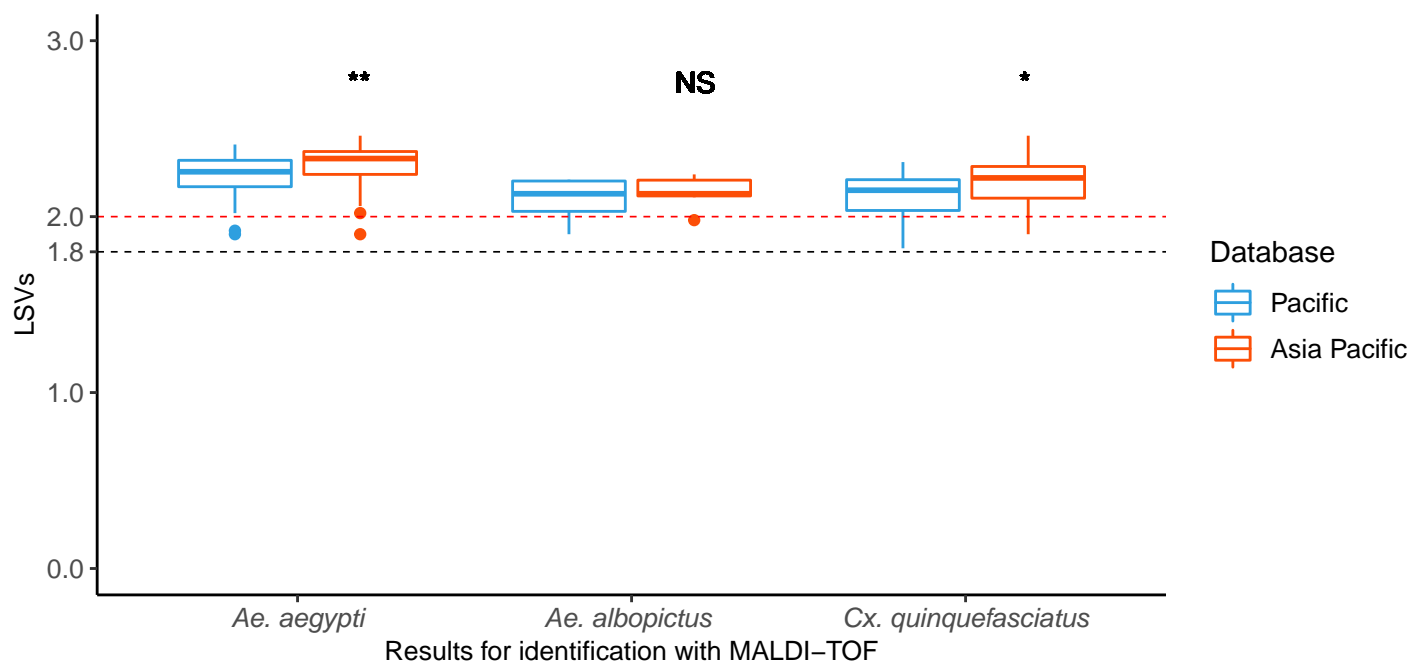

Supplement: S2 Fig — Three mosquito species were analyzed: Aedes aegypti (n = 40), Aedes albopictus (n = 8) and Culex quinquefasciatus (n = 19). Colors illustrate the database used in analysis. Black dashed-line corresponds to the threshold value for mosquito species identification, stated during previous study [29]. Red dashed-line corresponds to the threshold value for the identification of mosquito species belonging to the Scutellaris Group. Wilcoxon test, *p-value < 0.05, **p-value < 0.01. Abbreviation: NS, not significant. (PDF) [file pone.0276488.s002.pdf]

**A**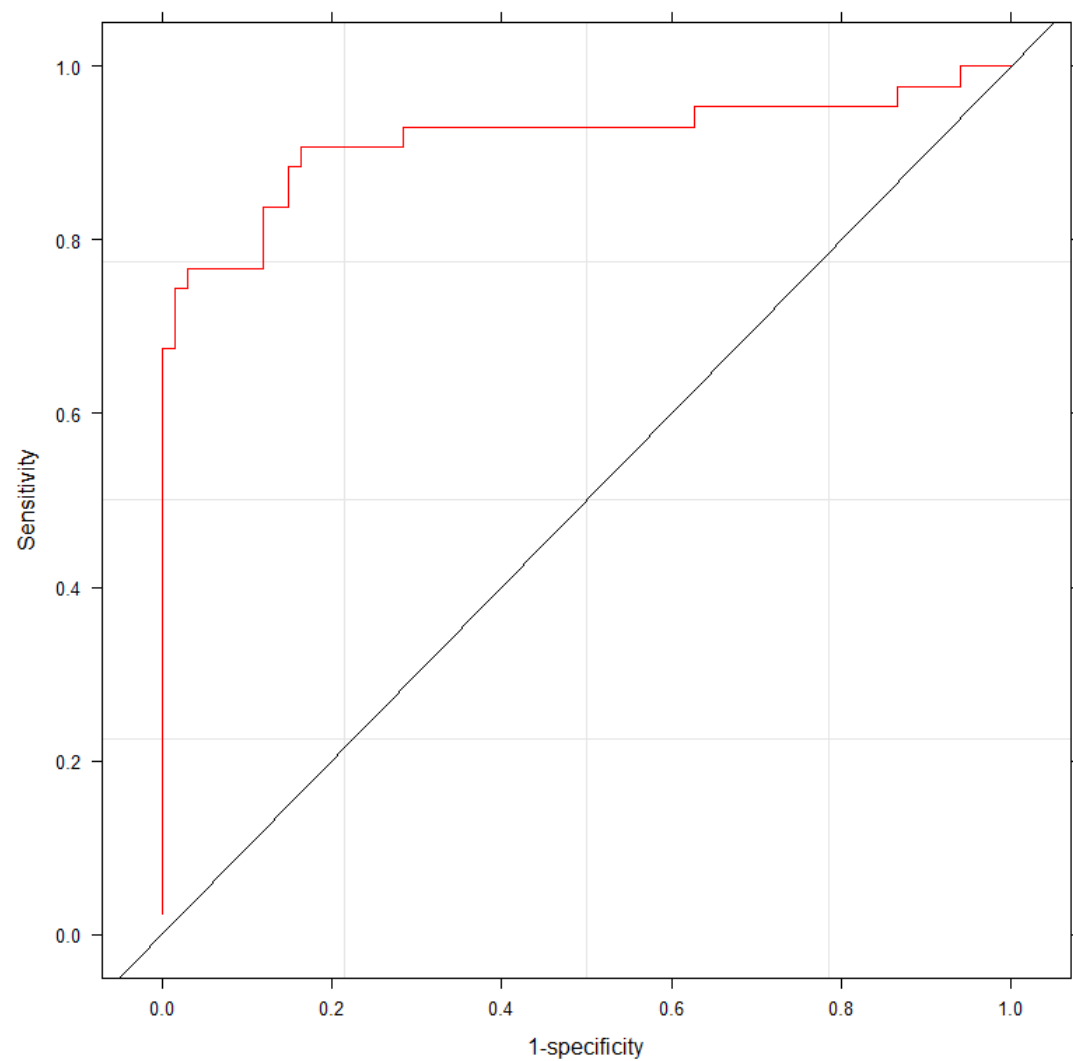**B**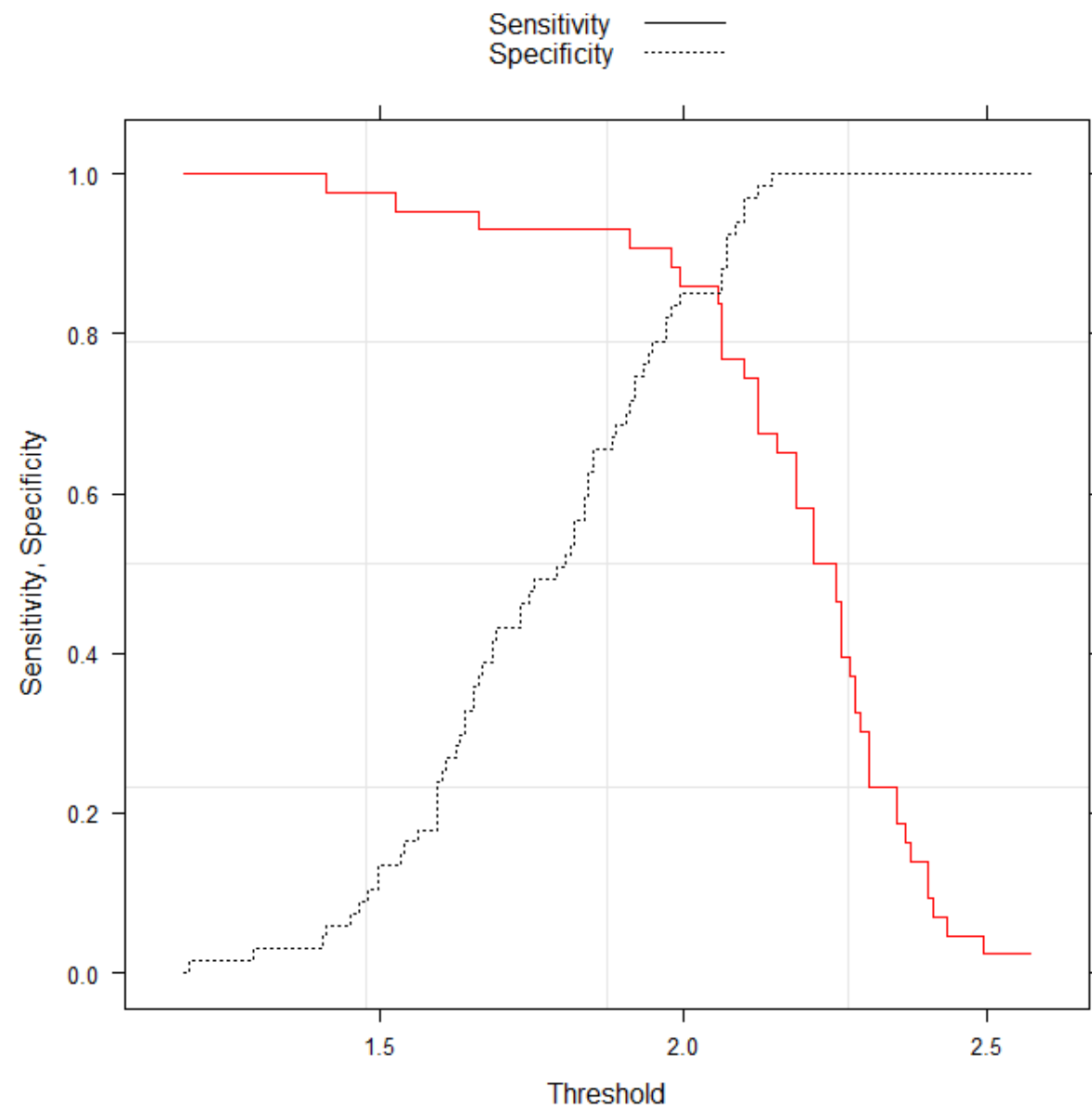

Supplement: S3 Fig — A- Representation of the AUC (Area under curve). B–Representation of the threshold value which maximizes the sensitivity and specificity of the technique. (PDF) [file pone.0276488.s003.pdf]

**A**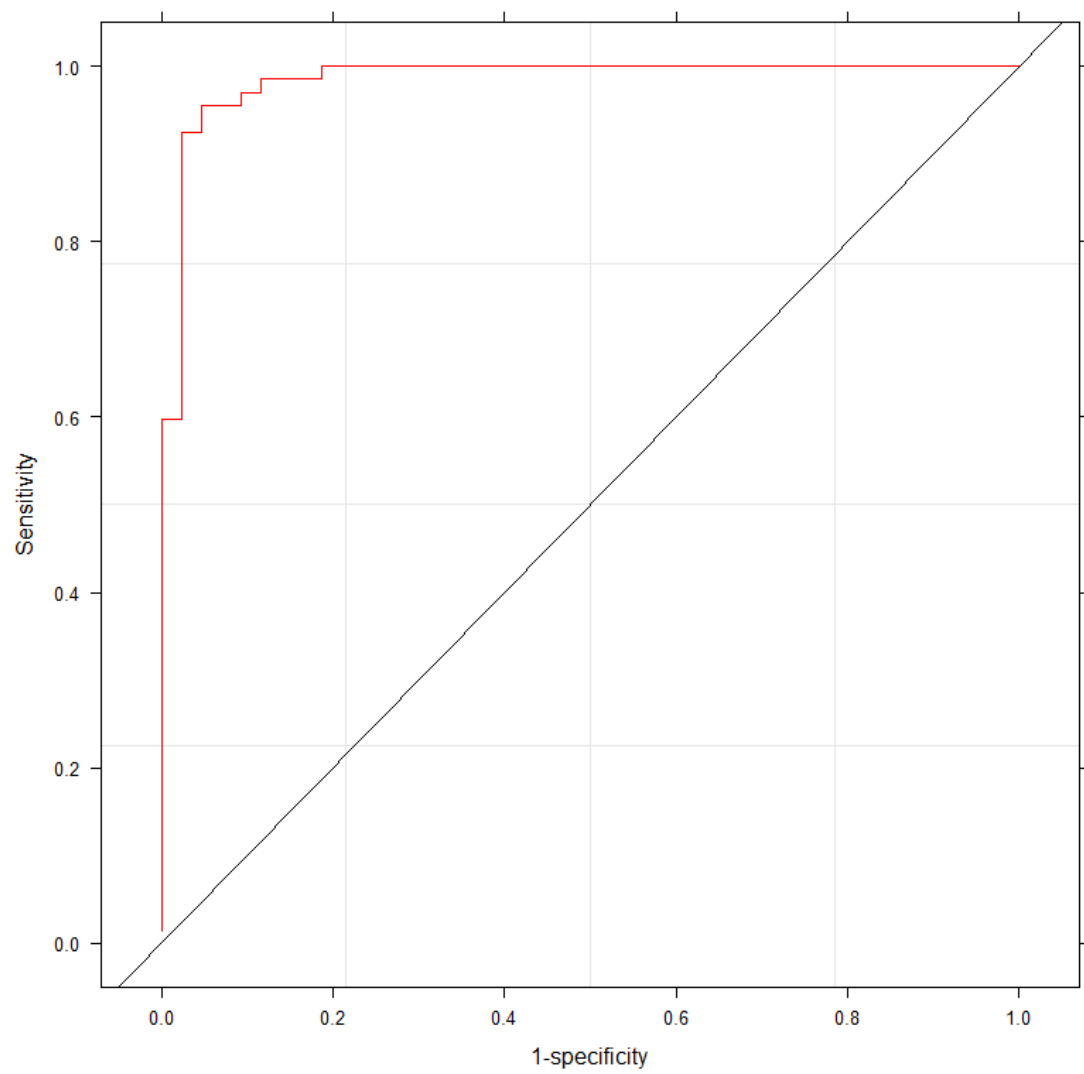**B**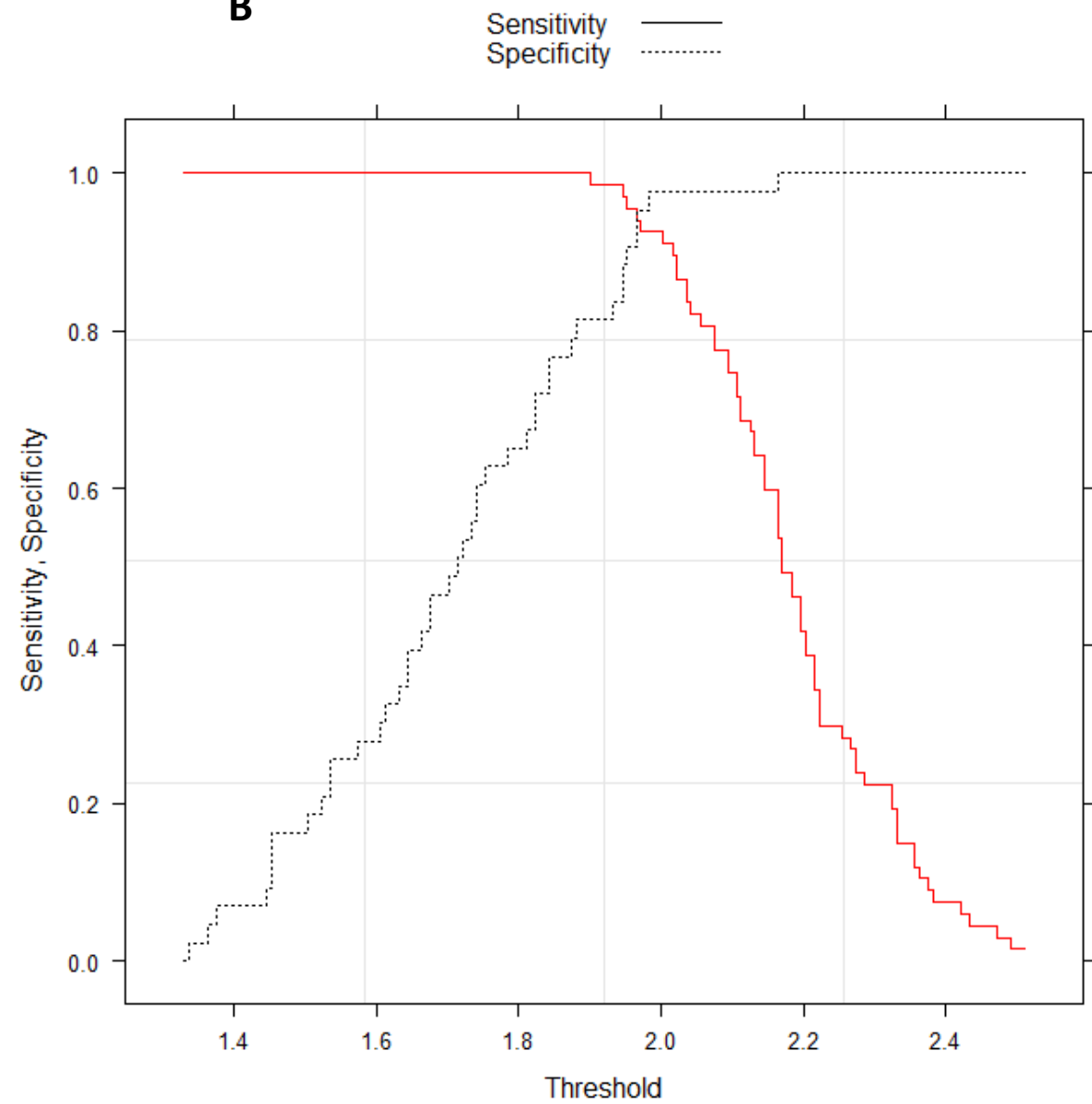

Supplement: S4 Fig — A- Representation of the AUC (Area under curve). B–Representation of the threshold value which maximizes the sensitivity and specificity of the technique. (PDF) [file pone.0276488.s004.pdf]
